# Characterization of highly frequent epitope-specific CD45RA^+^/CCR7^+/- ^T lymphocyte responses against p53-binding domains of the human polyomavirus BK large tumor antigen in HLA-A*0201+ BKV-seropositive donors

**DOI:** 10.1186/1479-5876-4-47

**Published:** 2006-11-10

**Authors:** Maurizio Provenzano, Laura Bracci, Stephen Wyler, Tvrtko Hudolin, Giovanni Sais, Rainer Gosert, Paul Zajac, Giorgio Palu', Michael Heberer, Hans H Hirsch, Giulio C Spagnoli

**Affiliations:** 1 Institute of Surgical Research and Hospital Management, University Hospital Basel, Switzerland; 2 Institutes for Medical Microbiology and Div. Infectious Diseases, University of Basel, Switzerland; 3 Department of Histology, Microbiology and Medical Biotechnologies, University of Padua, Italy; 4 Department of Cell Biology and Neurosciences, Istituto Superiore di Sanita', Rome, Italy; 5 Department of Urology, Clinical Hospital Center Zagreb, Croatia

## Abstract

Human polyomavirus BK (BKV) has been implicated in oncogenic transformation. Its ability to replicate is determined by the binding of its large tumor antigen (LTag) to products of tumor-suppressor genes regulating cell cycle, as specifically p53. We investigated CD8+ T immune responses to BKV LTag portions involved in p53 binding in HLA-A*0201+ BKV LTag experienced individuals.

Peptides selected from either p53-binding region (LTag_351–450 _and LTag_533–626_) by current algorithms and capacity to bind HLA-A*0201 molecule were used to stimulate CD8+ T responses, as assessed by IFN-γ gene expression ex vivo and detected by cytotoxicity assays following in vitro culture.

We observed epitope-specific immune responses in all HLA-A*0201+ BKV LTag experienced individuals tested. At least one epitope, LTag_579–587_; LLLIWFRPV, was naturally processed in non professional antigen presenting cells and induced cytotoxic responses with CTL precursor frequencies in the order of 1/20'000. Antigen specific CD8+ T cells were only detectable in the CD45RA+ subset, in both CCR7+ and CCR7- subpopulations.

These data indicate that widespread cellular immune responses against epitopes within BKV LTag-p53 binding regions exist and question their roles in immunosurveillance against tumors possibly associated with BKV infection.

## Background

Human polyomavirus BK (BKV) is a DNA virus belonging to the Polyomaviridae family that also includes human polyomavirus JC (JCV), and Simian virus 40 (SV40) (International Committee on the Taxonomy of Viruses (ICVD))[1]. The virus is ubiquitous in the human population, establishing latent infections in the kidney and the urogenital tract[2]. Spontaneous reactivation and low-level replication with shedding into urines is observed in 5–20% of healthy individuals[3]. Serological evidence indicates that nearly 90% of individuals are infected by early childhood, although a decrease in this rate during the human lifespan may be due to viral seroconversion (70–80%) [4,5]. Usually, polyomaviruses cause persistent subclinical infections in humans and BKV infection rarely leads to clinical manifestations. However, when the immune system is compromised, as following transplantation, HIV infection, chemotherapy or pharmacologic immunosuppression, rate and level of BKV replication increase and may lead to organ diseases[6]. The polyomavirus genome consists of the non-coding control region, the early genes and the late genes. The polyomavirus-encoded early gene product large tumor antigen (LTag) has been identified early on as a key regulatory molecule[7]. LTag interacts with a number of host cell molecules including the tumor-suppressor gene retinoblastoma family (Rb) products and p53. Initially, the LTag binds to products of the Rb-family (pRb, p107, and p130) thereby interfering with their activity and inducing the infected cell to enter the cell cycle (phase S). Subsequently, and most importantly, the LTag inactivation of p53 allows the re-phosphorylation of pRb through the cyclin-dependent kinase (cdk) pathway and prevents the p53-mediated cell apoptosis of infected cell[8].

This mechanism is used by the virus to keep the infected cells alive during productive infection but in non-permissive cells it may lead to cell transformation[9]. In fact, abortive replication may result in oncogenic transformation which has rendered polyomaviruses prototypes of DNA tumor virus well amenable to studies in experimental models. Indeed, p53 mutations and overall inactivation have been implicated in tumor progression.

The role of human polyomaviruses in human cancer is still debated. Recent investigations have associated them with the outgrowth of specific cancer types including colorectal cancer[10,11], glioblastomas[12,13], mesotheliomas[14], prostate cancer[15] and, possibly, lymphomas[16,17].

Since LTag mediated inactivation of p53 has been suggested to represent a critical step for viral oncogenicity[18,19], this antigen has been identified as an important target of cancer immunity in murine models[20]. The involvement of BKV LTag in the alteration of critical pathways of the human cell cycle together with the detection of LTag-p53 complexes by immunohistochemical analysis of normal and pathological specimens[15], prompted us to investigate the role of this viral antigen as target of cellular immune surveillance. The specific aim of this study was to evaluate in HLA-A*0201+ BKV LTag experienced donors immune responsiveness to candidate epitopes from portions of the LTag specifically expressed in long lasting infected cells and possibly playing a role in BKV mediated oncogenisis.

## Materials and methods

### Large Tag selection and peptide synthesis

The LTag sequence used in this study was selected by comparing 15 BKV LTag sequences available on the net[21] sharing a homology between 97 to 100% as detectable by using BLAST search[22]. Two major peptide-algorithms, BIMAS[23] and SYFPHEITI[24] were employed to list candidate immune dominant epitopes from LTag. A standardization of peptides' scores was applied to identify best binders according to their final values using the following formula: z_*i *_= [(x_*i*-_μ) σ] [25]

Four peptides were used as positive or negative controls: the HLA-A*0201-restricted vaccinia virus H_3_L peptide_184–192 _(SLSAYIIRV)[26], the HLA-A*0201-restricted MelanA/Mart1_26–35 _(ELAGIGILTV)[27], the HLA-A*2402-restricted CMV pp65_341–350 _(QYDPVALLFF) [28,29] and the HLA-A*0201-restricted CMV pp65_495–503 _(NLVPMVATV) [30]. Peptides were synthesized by Princeton Biomolecules (Langhorne, PA) with purity from 90 to 100% as analyzed by HPLC, dissolved in 100% DMSO and stored at -70°C until use.

### Peptide binding affinity to HLA-A*0201 processing defective T2 cells

HLA-A*0201+ human antigen processing defective T2 cells were diluted in PBS at a concentration of 2 × 10^6 ^cells/ml and were distributed in 96-well U bottom plates (100 μl total) in the presence or absence of peptides at a 250 μg/ml concentration. Plates were subsequently incubated for 16 hours at 37°C in humidified air containing 5% CO_2_. Cells were stained with FITC-tagged antibodies against human HLA-A*0201 (BD, Bioscience, San Jose, CA) and samples were analyzed by FACS. The fluorescence index (FI) was calculated by the following formula: (mean fluorescence experimental sample-mean fluorescence background)/mean fluorescence background). Results were reported in logarithmic scale with a 2-fold increase cut-off based on the fluorescence intensity in the presence of the irrelevant peptide HLA-A*2402-restricted CMV pp65_341–350_.

### Donor selection and serology

Upon informed consent, HLA-A*0201+ Caucasians (mean age: 36.8 ± 7.6) were selected for this study. Detection of BKV-specific Ab (IgG) in donor sera was performed by ELISA using recombinant LTag and capsid protein 1 (VP1) purified from baculovirus expression system (H.H.H. and R. G., unpublished data). Briefly, the N-terminal part of LTag overlapping with the small T-antigen was expressed as a Glutathione-S-transferase (GST)-fusion protein in SF9-insect cells and purified by gluthathione-sepharose affinity chromatography. The fusion proteins and GST were coated on microtiter plates according to standard procedures. Sera were diluted 1:400 and tested in duplicates at room temperature. After washing 5 times with PBS, peroxidase labeled goat anti-human IgG (1:10'000 dilution in PBS) were added for 30 min before extra washing (5 times with PBS). O-phenyldiamine (0.4 mg/L) was added as substrate for 30 min in the dark. The reaction was stopped with H_2_O_2 _and the OD450 nm was read. Reactive sera were defined by OD readings above background subtracted of GST+2SD.

### Peptide ex vivo induction and qrt-PCR analysis

Ex vivo induction of peptide-specific responses was attempted as follows. Briefly, PBMCs isolated from venous blood by Ficoll gradient centrifugation (Ficoll-hypaque density gradient), were incubated in 96 U bottom well plates at the concentration of 2 × 10^5 ^cells in 200 μl total RPMI medium supplemented with 100 μg/mL Kanamycin, 10 mM Hepes, 1 mM sodium pyruvate, 1 mM Glutamax and nonessential amino acids (all from GIBCO Paisley, Scotland) thereafter referred to as complete medium and 5% human serum (Blutspendezentrum, Kantonsspital Basel, Switzerland). After an overnight resting, cells were both peptide-stimulated (1 μM) or left unstimulated and 3 hours after they were harvested for RNA extraction (RNeasy^® ^Mini Kit Protocol, Qiagen, Basel, Switzerland) and cDNA synthesis (Invitrogen, Carlsbad, CA). Quantitative real-time PCR (qrt-PCR) assays were performed as previously described [31] and conducted on an ABI prism™ 7500 FAST sequence detection system using TaqMan^® ^Universal PCR Master Mix Reagents Kit (Applied Biosystems, Rotkreuz, Switzerland) and sets of primers and probes from cytokine genes (IFN-γ, IL-2, IL-4, and IL-10) already extensively utilized[28]. Beta actin (β-actin) was used as endogenous reference gene. Normalized data were subsequently presented as relative quantification. The 2^-ΔΔCt ^method [ΔΔC_T _= (C_T, _cytokine – C_T,_β-actin)_induction _– (C_T, _cytokine – C_T,_β-actin)_baseline, where _C_T _is the mean cycle times of the triplicate well readings] was utilized to compute the fold change of cytokine gene expression after peptide induction relative to baseline (unstimulated cells), normalized to an endogenous reference gene (β-actin)[32].

### Cell collection and culture

Subpopulations of cells (CD14+, CD8+, CD45RA+) were either positively or negatively purified from freshly isolated PBMCs by MACS (Miltenyi Biotech, Bergisch Gladbach, Germany) according to producers' protocols and in relation to different experimental procedures. For dendritic cell (DC) generation, isolated CD14+ were cultured for 5 to 7days in complete medium supplemented with 10% FCS (GIBCO Paisley, Scotland), β-mercaptoethanol 0.004%, recombinant human IL-4 (1000 UI/mL, courtesy of Dr. Lanzavecchia, Bellinzona, Switzerland) and recombinant human GM-CSF (50 ng/mL, Laboratorio Pablo Cassara', Buenos Aires, Argentina) to generate immature DC (iDCs). Maturation of iDCs was induced by exposure to lipopolysaccaride (*Abortus Aequi*, Sigma-Aldrich, St. Louis, MO) at a concentration of 1 μg/mL. Both whole CD8+ and CD45RA+ purified T cells were subsequently plated in complete medium supplemented with 5% human serum in either 96-well U bottom or 24-well plates for in vitro inductions at various concentrations and time exposures. Mature peptide-loaded DCs (mDCs) were used as APC either for priming or for rounds of restimulations. Recombinant human IL-2 (rhIL-2) was administered to the cultures at different concentrations according to experimental procedures.

### Cytotoxic T lymphocytes in vitro expansion and cytotoxicity assay

CD8+ T cells purified from PBMCs of healthy donors (10^6^/mL) were primed with irradiated (30 Gy) autologous mDCs (2 × 10^5^/mL) previously loaded for 2 hours with peptide at a final concentration of 5 μg/mL. On days7 and 14 cultures were restimulated with peptide loaded mDCs. During the in vitro induction the cells were supplemented every other day with rhIL-2 at the final concentration of 20 UI/mL. Cytotoxic activity was tested on day 21 by using a 4-hrs chromium release assay on T2 cells previously labeled with ^51^Cr (50 μCi of ^51^Cr for 1 h at 37°C) and pulsed for 2hours with cognate or control peptides at a concentration of 2.5 μg/mL in triplicate wells. Specific lysis of target cells was calculated according to the standard formula: 100 × [(cpm experimental release - cpm spontaneous release)/(cpm maximal release - cpm spontaneous release)]. Following testing, cytotoxic T lymphocytes (CTLs) were expanded by repeated restimulations to generate epitope-specific CTL lines which were used to test functional activities and to analyse natural processing of defined peptides.

### Limiting dilution analysis (LDA)

CD8+ T cells were cultured in 96-well plates according to the same conditions as outlined above at 10'000, 5'000 and 2'500 cells/well for total of 32 wells for each concentration. Autologous mDCs were pulsed with peptides, irradiated and used as APC at a final concentration of 2'500 cells/well. On day 7 cells were re-stimulated following the same procedure. On day 3 and day 10, rhIL-2 was administered to cultures at a concentration of 20 UI/ml and 100 UI/ml, respectively. Cytotoxic activity was tested on day 14 against peptide pulsed ^51^Cr-labeled T2 cells, as reported above. Cytotoxic T cell precursor (CTLp) frequencies were evaluated as previously described[33]. Wells were considered positive when their cytolysis exceeded 3 standard deviations and at least 12% above average values of negative control lysis. On the basis of the single-hit Poisson model, the frequency of antigen specific CTLp's was estimated from the initial responder cell number at which 37% of the wells were negative for cytotoxicity.

### Plasmid generation and transfection of HLA-A*0201 target cells

In plasmid DNA pTRE2LTag, the cDNA coding sequence of LTag of BKV is under control of a tetracycline-controlled transactivator-dependent promoter. The intron of the LTag was removed by 'gene splicing' by overlap extension[34]. Two DNA fragments were generated by PCR using primer pairs 1 -2: (1) 5'-GAGAGAGCTAGCCACCATGGATAAAGTTCTTAACAGGGAAGA-3' (2) 5'-TTCTGTTCCATAGGTTGGCACCTCTGAGCTACTCCAGGTTCC-3' and 3–4: (3) 5'-GGAACCTGGAGTAGCTCAGAGGTGCCAACCTATGGAACAGAA-3' (4) 5'-GAGAGAATCGATTATTTTGGGGGTGGTGTTTTAGG-3' on DNA extracted from BKV-positive urine as template. The two DNA fragments were subsequently joined in a PCR reaction containing primer pair 1–4. The amplification product was digested with *Cla*I and *Nhe*I, followed by ligation into *Cla*I-*Nhe*I digested pTRE2pur (BD Biosciences, San Jose, CA). The integrity of plasmid DNA was verified by automated sequencing on a Beckman CEQ 8000 (Beckman Coulter Inc. Fullerton, CA).

The metastatic melanoma HBL cell line expressing HLA-A*0201 molecule were selected for pTRE2LTag transfection. Cells were routinely passaged in conventional cultures in complete medium supplemented with 10% heat-inactivated FCS. Transfection of HBL cells was performed by liposome-mediated gene transfer in 6 or 12-well plates. Cells at 90–95% confluency were transfected with a plasmid DNA (1.6 and 3.2 μg) to Lipofectamine 2000 (2 and 3 μl) (Invitrogen, Carlsbad, CA) ratio of 1.06 according to the manufacturer's recommendations. For transfection, pTRE2LTag and pTet-Off (BD Biosciences, San Jose, CA) were mixed at a ratio of 1:1. At 24 hours post transfection, the cells were transferred into T75 culture flasks. To determine the peak of protein expression, a variant of wild-type green fluorescent protein plasmid (pEGFP-N1; BD Biosciences, San Jose, CA) was transfected into parallel cell cultures and the enhanced green fluorescent protein (EGFP) expression was monitored by fluorescence microscopy at 24 and 48 hours post transfection. The LTag gene expression was measured by qrt-PCR using the following primers and probe[15]:

Forward: 5'-TTTTGGAACCTGGAGTAGCTCAGAGGTTT-3'

Reverse: 5'-GCTTGACTAAGAAACTGGTGTAGAT-3'

Probe: 5'-TTGAGTGTTGAGAATCTGCTGTTGCTTCTTCATCACTGGCAAACA-3'

### Standard enzyme-linked immunosorbent assay

Release of IFN-γ protein by either in vitro sensitized PBMCs after peptide re-stimulation or expanded CTLs cultured with pTRE2LTag BKV tranfected targets was measured using an ELISA kit (Endogen, Woburn, MA) on culture supernatants collected 18 hours after the last induction. ELISA results were extrapolated from a standard curve generated by linear regression. All assays were performed in duplicate and results were reported as average values.

### Cell phenotype and multimer staining analysis

HLA-A*0201 MHC Pro5™ PE-labeled pentamers for LTag_579–587_, LTag_406–414 _and Melan-A/MART-1_26–35 _(ProImmune, UK) were used for surface staining of the cells under investigation. Samples were analyzed on a FACSCalibur flow-cytometer equipped with Cellquest software (Becton Dickinson, San Jose, CA). Cells were first stained with 1 μl MHC PE-labeled pentamer for 15 min at 4°C in the dark. Subsequently, they were washed once and stained with 5 μl of CD8-PE-Cy7, CCR7-APC and CD45RA-FITC (BD, Bioscience, San Jose, CA) for 30 min at 4°C (on ice) in the dark. After staining, cells were washed twice and immediately analyzed on a flow cytometer or fixed in 200 μl 4% paraformaldehyde in PBS solution, kept at 4°C in the dark and analyzed later.

## Results

### Prediction of HLA-A*0201-restricted T cell epitopes within BKV LTag

BIMAS and SYFPEITHI algorithm programs were used to select HLA-A*0201 binding nonamer peptides from BKV LTag. An unbiased search was performed focusing in particular on p53-binding regions of LTag. Standardization of algorithm-scores[25] led to the final selection of 14 peptides out of a total of 250 sorted by the programs (Tab 1). Among the 14 peptides selected, 6 were nested within the LTag p53-binding domain 351–450 (LTag_351–450_) and 3 within the LTag p53-binding domain 533–626 (LTag_533–626_). No peptide was selected within the LTag p300-binding domain 1–82 (LTag_1–82_) and the LTag pRb- (p107-) binding domain 101–118 (LTag_101–118_). The remaining 5 peptides were randomly nested within LTag regions not presumed to be directly involved in the regulation of the cell cycle (Tab 2 and Tab 3)[35].

**Table 1:** Prediction of HLA-A*0201 restricted T cell epitopes within BKV LTag

***starting position***	***peptide sequence***	***BIMAS value***	STND score*	***SIPHEYTI value***	STND score*
410	**FLHCIVFNV**	4267.99	15.274	25	2.232
216	**KLCTFSFLI**	804.786	2.7922	20	1.272
406	**VIFDFLHCI**	323.333	1.057	25	2.232
199	**FLTPHRHRV**	319.939	1.0448	23	1.848
398	**CLLPKMDSV**	290.025	0.937	27	2.616
176	**KLMEKYSVT**	243.333	0.7687	19	1.079
579	**LLLIWFRPV**	166.243	0.4909	24	2.04
157	**TLACFAVYT**	155.747	0.453	19	1.079
362	**MLTERFNHI**	138.162	0.3896	20	1.272
558	**SLQNSEFLL**	123.902	0.3383	22	1.656
570	**ILQSGMTLL**	83.527	0.1927	24	2.04
395	**WLHCLLPKM**	52.561	0.0811	22	1.656
436	**TLAAGLLDL**	49.134	0.0688	29	3
472	**VVFEDVKGT**	38.676	0.0311	16	0.503

*Sequences selected using the standardization method

**Table 2:** Peptide nomenclature according to their starting position in the LTag

LTag 157	TLACFAVYT
LTag 176	KLMEKYSVT
LTag 199	FLTPHRHRV
LTag 216	KLCTFSFLI
LTag 362	MLTERFNHI
LTag 395	WLHCLLPKM
LTag 398	CLLPKMDSV
LTag 406	VIFDFLHCI
LTag 410	FLHCIVFNV
LTag 436	TLAAGLLDL
LTag 472	VVFEDVKGT
LTag 558	SLQNSEFLL9
LTag 570	ILQSGMTLL
LTag 579	LLLIWFRPV

**Table 3:** Selected sequences as they nest in the LTag domains

p300 domain 1–82	MDKVLNREESMELMDLLGLERAAWGNLSLMRKAYLRKCKEFHPDKGGDEDKMKRMNTLYKKMEQDVK
pRb domain 101–118	VAHQPDFGTWSSSEVPTYGTEEWESWWSSFNEKWDEDLFCHEDMFASDEEATADSQHSTPPKKKR
	KVEDPKDFPCDLHQFLSQAVFSNR**TLACFAVYT**TKEKAQILYK**KLMEKYSVT**FISRHMCAGHNIIF
DNA binding	**FLTPHRHRV**SAINNFCQ**KLCTFSFLI**CKGVNKEYLLYSALTRDPYHTIEESIQGGLKEHDFNPEEPEETKQVSW
	KLITEYAVETKCEDVFLLLGMYLEFQYNVEECKKCQKKDQPYHFKYHEKHFANATIFAESKNQKSICQQAVDTVLAKK
p53 domain 351–450	RVDSLHMTREE**MLTERFNHI**LDKMDLIFGAHGNAVLEQYMAGVA**WLHCLLPKMDSVIFDFLHCIVFNV**
	PKRRYWLFKGPIDSGKT**TLAAGLLDL**CGGKAL
ATPase activity	NVNLPMERLTFELGVAIDQYM**VVFEDVKGT**GAESKDLPSGHGINNLDSLRDYLDGSVKVNLEKKHLNKRTQIFPP
	GLVTMNE
p53 domain 533–626	YPVPKTLQARFVRQIDFRPKIYLRK**SLQNSEFLL**EKR**ILQSGMTLLLLLIWFRPV**ADFATDIQSRIVEWKERLD
	SEISMYTFSRMKYNICMGKC
	ILDITREEDSETEDSGHGSSTESQSQCSSQVSDTSAPAEDSQRSDPHSQELHLCKGFQCFKRPKTPPPK

**Figure 1:**
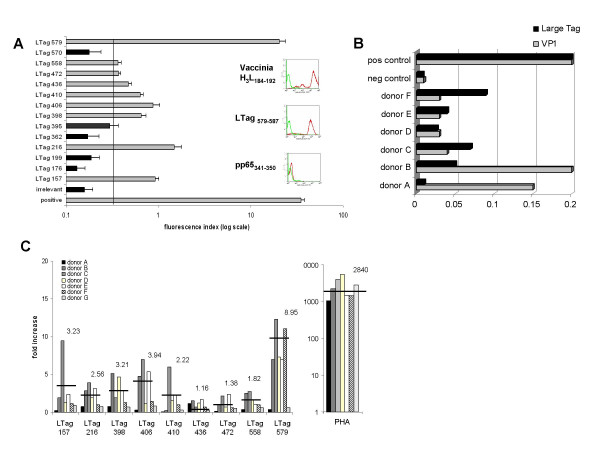
HLA-A*0201 binding and immunostimulatory capacity of peptides from BKV LTag sequence. Nonamer peptides identified by standardized assessment of epitope finding algorithms within BKV LTag sequence were tested for MHC affinity by T2 binding assay (panel A). Data are reported as average mean fluorescence intensities from three different experiments. Representative histograms obtained upon incubation of T2 cells in the presence of vaccinia H_3_L_184–192 _(positive control), pp65_341–350 _(irrelevant peptide) and LTag_579–587 _(LTag peptide best binder) are also shown. Panel B shows data related to BKV antigen specific serological response to both LTag and VP1 antigen in the group of donors included in the study. Panel C shows responsiveness of PBMCs from BKV LTag seropositive donors cultured in the presence of the indicated peptides. The capacity to induce IFN-γ gene expression upon stimulation ex vivo was analysed by qrt-PCR. Results are depicted as IFN-γ relative gene expression (relative quantification, 2^-ΔΔCt ^method) as compared to unstimulated PBMCs and include data from all HLA-A*0201+ BKV LTag seropositive donors tested (B to F), including both HLA-A*0201+ BKV LTag seronegative (donor A) and HLA-A*0201 negative (donor G) controls. The results are representative of two independent experiments.

To validate algorithm predictions, we performed a HLA-A*0201 T2 binding assays to evaluate the ability of individual peptides within LTag_351–450 _and LTag_533–626 _regions to stably interact with HLA-A*0201 determinants over time (16 hours). HLA-A*2402 CMV pp65_341–350 _(QYDPVALLFF) was used as irrelevant negative control peptide while vaccinia virus H_3_L_184–192 _peptide (SLSAYIIRV) was used as positive control. Based on these studies, four peptides within p53-binding region LTag_351–450 _(LTag 398, 406, 410, and 436), two within p53-binding region Tag_533–626 _(LTag 558 and 579) and three from non p53-binding regions (LTag 157, 216 and 472) capable of increasing * 2-fold the HLA-A*0201 specific mean fluorescence intensity on T2 cultured in the presence of the irrelevant negative control peptide were identified (Figure 1A).

### Ex vivo immune reactivation against HLA-A*0201+ BKV LTag experienced donors

PBMCs were obtained from seven donors (A to G). Five of them (B to F) were HLA-A*0201+ and showed evidence of humoral response against BKV LTag. Donor A was HLA-A*0201+ but BKV LTag seronegative (Figure 1B), while donor G was HLA-A*0201 negative.

Cells were ex vivo stimulated with 1 μM of each of the 6 LTag p53-binding regions peptides selected upon combining algorithm data and T2 binding assays (LTag 398, 406, 410, 436, 558, and 579). The three peptides not included in the p53-binding domains (LTag 157, 216, and 472) were also tested to investigate eventual immune responses against determinants encompassed by BKV LTag regions not presumably involved in the cell cycle. Responsiveness was assessed by using qrt-PCR to evaluate fold increase of IFN-γ gene expression in peptide stimulated cultures as compared to unstimulated controls. A threshold of 2-fold increase over the baseline was considered as a cut-off. LTag 579 induced the highest IFN-γ gene expression in PBMCs from all the 5 HLA-A*0201+ BKV LTag seropositive donors tested (donors B-F). Potential immunogenicity of the p53 binding domain LTag_533–626 _was confirmed by the response induced by LTag 558 in two of these donors. Ex vivo immune responses against LTag 398, 406, and 410 peptides from p53 binding region LTag_351–450 _were also detected in 4/5 donors. Furthermore, immune reactivation was also induced by LTag 157, 216, and 472 peptides, particularly in PBMCs from one of the donors included in this group (donor C) (Figure 1C). Importantly, LTag 436, while showing effective T2 binding, was unable to induce specific IFN-γ gene expression in any of the donors under investigation. As expectable, no responsiveness was detectable in cells from HLA-A*0201+ donor A, seronegative for BKV LTag, nor in cells from HLA-A*0201 negative donor G.

### Epitope-specific cytotoxic activity by BKV LTag_351–450 _and LTag_533–626 _in vitro expanded CD8+ T cells

A 3-week peptide-specific in vitro expansion was carried out on isolated CD8+ T lymphocytes from the five HLA-A*0201+ BKV LTag seropositive donors included in the study (B through F). The eight candidate immunogenic LTag derived peptides identified in the previous phase were used for the induction of cytotoxic activity in CD8+ T cells from donors shown to be responsive in terms of specific "ex vivo" IFN-γ gene expression upon cognate peptide stimulation. ^51^Cr release cytotoxicity assays were used as read-out.

**Figure 2:**
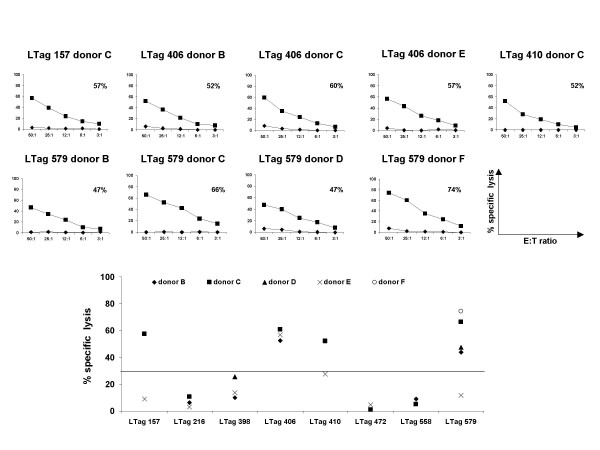
Detection of CTL activity against epitopes from BKV LTag domains among HLA-A*0201+ healthy donors. CD8+ T lymphocytes from five HLA-A*0201+ BKV LTag seropositive donors (B through F) were expanded in vitro for 3 weeks, upon weekly stimulation using autologous irradiated mDCs loaded with BKV LTag derived peptides selected by combining results from T2 binding and IFN-γ gene expression assays (≥ 2-fold, as compared to either irrelevant peptide or unstimulated cells). Cytotoxic activity was then tested against specific (squares) or control (diamonds) peptide-loaded targets. Specific cytotoxicity values at 50:1 E:T ratio are also included in each panel. An overview of specific lysis for all peptides tested in all BKV LTag seropositive responders at 50:1 E:T ratio is reported in the lower panel, where an arbitrary cut-off of 30% was applied.

LTag 579 peptide induced specific cytotoxic activity in cells from 4/5 responders. LTag 406 did in 3/3, LTag 410 and LTag 157 in 1/2 each (Figure 2). In contrast, LTag 216, LTag 398, LTag 472 and LTag 558, while inducing some degree of specific IFN-γ gene expression "ex vivo", were unable to stimulate the generation of peptide specific cytotoxic activity.

**Figure 3:**
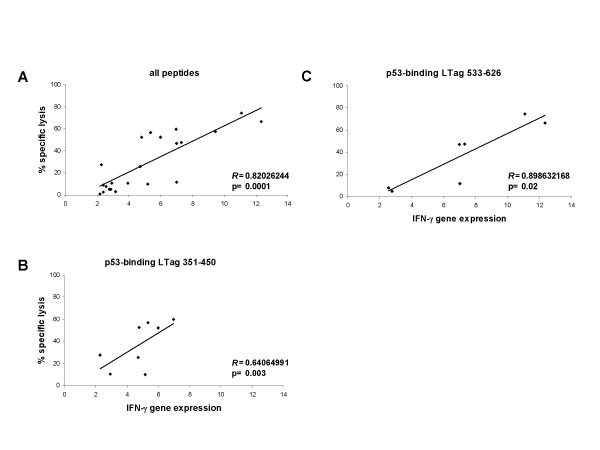
**Correlation between cytokine gene expression and lytic activity in T cells stimulated by BKV LTag epitopes**. Panels display correlations between IFN-γ gene expression, reported as fold increase, as compared to unstimulated cells, and specific cytotoxic activity as detectable upon 3-week in vitro culture at a 50:1 E:T ratio. Correlations were significant either when overall responses to LTag epitopes were analyzed (Pearson's *R *= 0.820, p = 0.0001, panel A) or when responses to both p53-binding domain LTag_351–450 _(Pearson's *R *= 0.640, p = 0.003, panel B) and LTag_533–626 _(Pearson's *R *= 0.898, p = 0.02, panel C) were separately evaluated. These data firmly establish that the ability of discrete peptides to induce specific cytotoxicity can be reliably predicted based on their degree of "ex vivo" stimulatory capacity regarding IFN-γ gene expression.

To further validate the specific CTL response obtained against the p53-binding regions LTag_351–450 _and Tag_533–626_, we correlated the extent of "ex vivo" immune reactivation detected after a 3-hour peptide-induction and the cytotoxic responses detectable upon a 3-week in vitro culture at a 50:1 E:T ratio. A highly significant correlation between the extent of IFN-γ gene expression and the percent specific lysis elicited respectively by 3-hrs ex vivo and 3-week in vitro stimulated CD8+ T cells with all peptides selected from all five HLA-A*0201+ BKV LTag seropositive responders tested was indeed observed (Pearson's *R *= 0.820, p = 0.0001, Figure 3A). Correlations were still highly significant when responses against specific p53-binding domains were analyzed individually (LTag_351–450_; Pearson's *R *= 0.640, p = 0.003 and LTag_533–626_; Pearson's *R *= 0.898, p = 0.02) (Figure 3B and 3C).

### HLA-A*0201-restricted LTag 579 epitope is a naturally processed immune determinant among p53-binding regions LTag_351–450 _and LTag_533–626_

**Figure 4:**
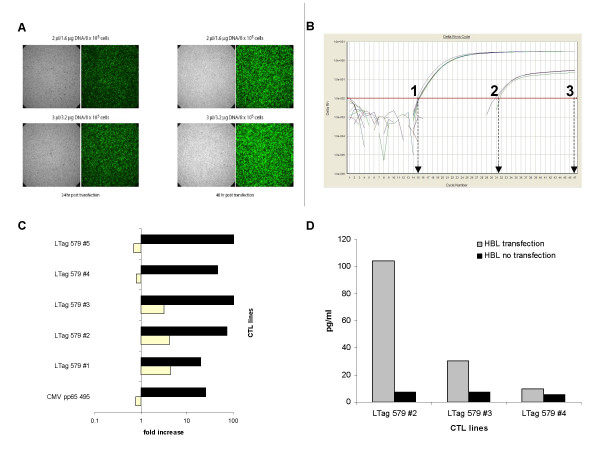
HLA-A*0201+ BKV LTag transfected target cells are recognized by LTag_579–587 _specific CTLs. HLA-A*0201+ HBL cells at 90–95% confluency were transfected by liposome-mediated gene transfer with plasmid DNA pTRE2LTag and pTet-Off (mixed at 1:1 ratio) and peaks of cell permissibility to plasmid were monitored by using a parallel pEGFP-N1 transfected cell culture as analysed by fluorescence microscopy at 2 different plasmid concentrations at 24 and 48 hours after transfection (panel A). Quantitative rt-PCR transient LTag gene expression showing means of Ct value of 31 for HBL transfected cell (2) and Ct value of 47 for untransfected cell (3), as compared to β-actin Ct values of 15 for either HBL transfected or untransfected culture (1) is also reported in panel B. Panel C reports fold increases of IFN-γ gene expression in five LTag 579 in vitro expanded CTL lines upon stimulation with transfected HBL (white bars) as compared with autologous peptide (LTag 579)-loaded APC (black bars). Results refer to IFN-γ relative gene expression (2^-ΔΔCt ^method) related to either untransfected HBL or non peptide-loaded APC. Panel D reports amounts of IFN-γ protein released by LTag 579 specific CTLs following culture in the presence of transfected (grey bars) or untransfected cells (black bars).

The melanoma cell line HBL expressing the HLA-A*0201 molecule was transfected with BKV LTag and used as target to test the ability of specific epitopes to be naturally processed and presented in the context of the HLA-A*0201 restriction. The highest transgene expression efficiency was achieved 48 hours after transfection, as suggested by detection of EGFP by fluorescence microscopy (Figure 4A). The transient LTag expression after 48 hours was also confirmed at the gene level with similar timing (Figure 4B).

LTag 579-expanded CTL lines previously tested for their ability to lyse LTag 579-loaded T2 cells (90% to 97% at 50:1 E:T ratio; data not shown) were incubated at a 5:1 E:T ratio with either LTag transfected or untransfected HBL cells. The resulting antigen specific activation was evaluated after 3-hrs incubation as IFN-γ gene expression and after 18-hrs incubation as IFN-γ protein release. A CMV pp65_495–503 _CTL line generated from a CMV seropositive subject was used as a control. Three out of five LTag 579-expanded CTL lines tested expressed IFN-γ gene upon stimulation by LTag transfected cells, although to a lower extent as compared to induction by peptide loaded autologous APC. (Figure 4C). Two LTag 579-expanded CTL lines consistently released the corresponding protein (Figure 4D). On the other hand, LTag 406 and 410 stimulated CTLs failed to specifically recognize LTag tranfected targets, as detectable by IFN-γ gene expression and protein production (data not shown).

### Epitope-specific cytotoxic T cell precursor frequency for the immunodominant determinant LTag 579 within BKV LTag_533–626_

**Figure 5:**
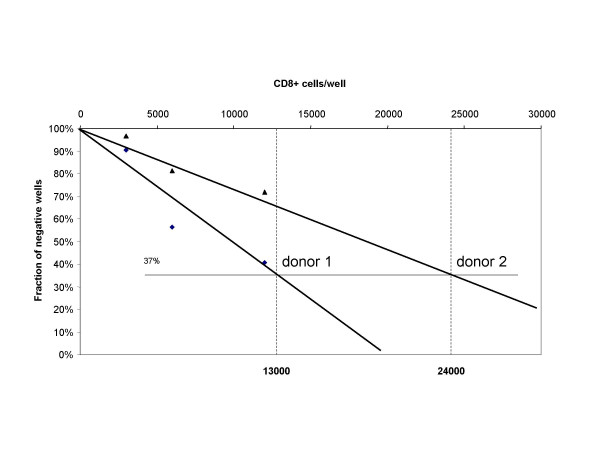
Frequency of LTag_579–587 _peptide-specific CTLp in HLA-A*0201+ BKV LTag seropositive donors. Frequency of LTag 579 peptide-specific CTLp from two HLA-A*0201+ BKV LTag seropositive donors was evaluated by LDA, as detailed in "Materials and methods", taking advantage of Poisson distribution analysis.

T cell precursor (CTLp) frequency for LTag 579 was then evaluated in HLA-A*0201+ LTag BKV seropositive donors by limiting dilution analysis (LDA), according to the Poisson distribution as described in "materials and methods". The LTag 579 specific CTLp frequency under cognate peptide stimulation, as detectable in two representative donors, was of 1:13'000 and 1: 24'000 (Figure 5).

### Ex vivo analysis of BKV LTag 579 specific T cell population

**Figure 6:**
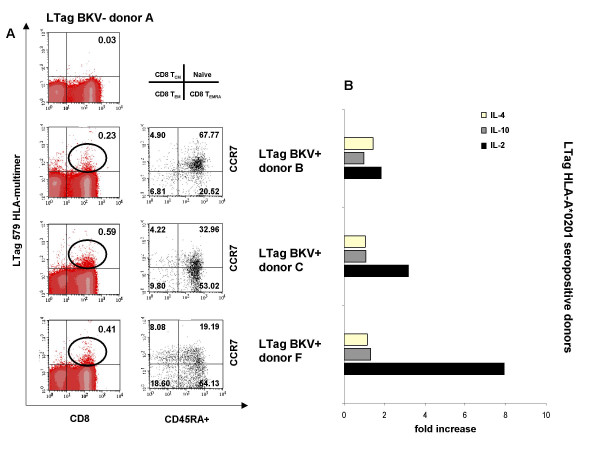
Phenotype and functions of LTag 579/HLA-A*0201 multimer-labeled specific T cells "ex vivo". PBMCs from three HLA-A*0201+ BKV LTag seropositive and one HLA-A*0201+ BKV LTag seronegative donors were stained with LTag 579/HLA-A*0201-multimers and the ex vivo CD8+ T cell phenotype was analyzed. Panel A shows frequencies of specific LTag 579-HLA-A*0201 multimer positive cells plotted against CD8+ T cells: cells were considered positive if their mean fluorescence intensities exceeded by ten fold that of multimer negative CD8+ cells. LTag 579/HLA-A*0201+ cells were also evaluated for their CD45RA and CCR7 surface expression. Panel B reports the expression of IL-2, IL-4, and IL-10 genes by "ex vivo" peptide induced PBMCs from the same donors. Data are reported as fold increase of specific gene expression (relative quantification, 2^-ΔΔCt ^method), as compared to unstimulated cultures.

CD8+ T cell phenotype of LTag 579 epitope-specific cells was then analysed ex vivo in three HLA-A*0201+ BKV LTag seropositive and one HLA-A*0201+ BKV LTag seronegative donors. LTag 579-HLA multimer specific CD8+ T cells frequency for HLA-A*0201+ BKV LTag seropositive donors was of 0.23%, 0.59% and 0.41%, respectively. Epitope specific CD8+ populations showed a prevailingly CD45RA positivity consistent with a naïve CD45RA+, CCR7+ phenotype and an effector-memory CD45RA+, CCR7- phenotype (Figure 6A). The gene expression of three cytokines implicated in the control of CD8+ T cell immune reactivity (IL-2, IL-4, and IL-10) was also studied ex vivo. LTag 579 induced the expression of IL-2 in two out of the three donors, whereas expression of IL-4 and IL-10 was always negligible (Figure 6B).

### Characterization of the T cell population stimulated by BKV LTag 579

**Figure 7:**
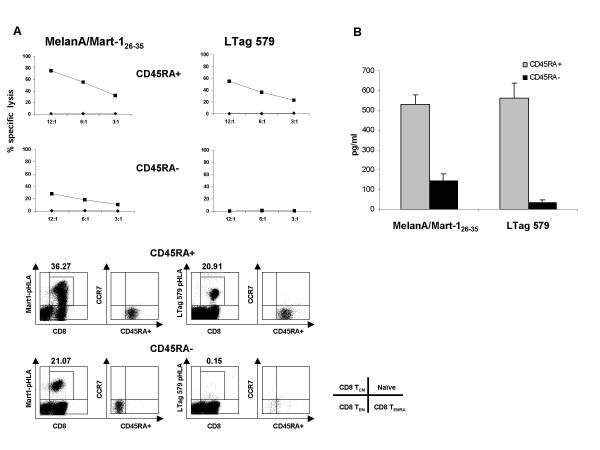
**BKV LTag 579 specific CD8+ T cells belong to the CD45RA+ subpopulation**. CD8+ T cells from HLA-A*0201+ BKV LTag seropositive donors were separated into CD45RA+ and CD45RA- fractions prior to stimulation with HLA-A*0201 restricted melanoma associated antigen MelanA/Mart-1_26–35 _or BKV LTag 579 peptide. After 3-weekly restimulations in the presence of peptide-loaded autologous mature DCs, cytotoxic activity was tested by ^51^Cr release assays, using as targets T2 cells following pre-incubation in the presence of specific or control peptides. The phenotypes of CD8+ cells stained by MelanA/Mart-1_26–35 _or BKV LTag 579 specific HLA-A*0201 multimers were also evaluated (panel A). Peptide specific IFN-γ production elicited by CD45RA+ and CD45RA- CD8+ T cell subpopulations is reported on panel B.

Prompted by these data, we then attempted a further characterization of LTag 579 specific HLA-A*0201-restricted CTLs. CD8+ T cells were separated into CD45RA+ and CD45RA- fractions prior to peptide stimulation. The HLA-A*0201-restricted melanoma associated antigen MelanA/Mart-1_26–35 _peptide was used as control due to its ability to prime and expand specific T cell with a prevailingly CD45RA+ phenotype in healthy donors[36]. After a 3-week induction by LTag 579 or MelanA/Mart-1_26–35 _peptides, cells from the CD45RA+ fraction showed a high cytotoxic activity against either epitope under investigation. Specific lysis (SL) was still detectable at a 3:1 E:T ratio (MelanA/Mart-1_26–35 _ΔSL_3:1 _= 32.06; LTag 579 ΔSL_3:1 _= 23.19). While low level MelanA/Mart-1_26–35 _specific cytotoxic activity was still observed in stimulated cultures generated from CD45RA- CD8+ T cell (MelanA/Mart-1_26–35_ΔSL_12:1 _= 28.17%), we were unable to generate CTL specific for LTag 579 from this cellular fraction (Figure 7A, upper panel). FACS analysis conducted on MelanA/Mart-1_26–35 _(36.27%) and LTag 579 (20.91%) HLA-A*0201 specific multimers revealed that over 99% of specific CD8+ T cells indeed displayed a late effector phenotype T_EMRA _(CD45RA+, CCR7-)[36] (Figure 7A, lower panel). Notably, IFN-γ protein release assays carried on supernatants of MelanA/Mart-1_26–35 _or LTag 579 in vitro expanded cells confirmed the finding that specific immune responses were prevailingly inducible in the CD45RA+ derived fraction (Figure 7B)

### BLAST search for specific homologies of p53-binding LTag 579

In order to address the possibility of cross-reactivities between epitopes encompassed by the two human polyomaviruses BK and JC, we analyzed possible homologies within all human polyomavirus strains for the p53-binding BKV region (LTag_533–626_) nesting LTag_579–587 _LLLIWFRPV, also including SV40. We noted that this region shares a homology between 98 to 100% with both BK and JC LTag in almost 100% of the strains analyzed. In particular, a single amino acid substitution in position 6 involving serine *vs *phenylalanine (Ser→Phe) was found in one out of 15 BKV strains and in position 7 involving lysine *vs *arginine (Lys→Arg) in one out of 155 JCV strains screened. This homology decreases to about 70% in comparison to the SV40 large Tag since all the 13 SV40 strains screened report substitutions in positions 2 involving methionine *vs *leucine (Met→Leu) and position 6 involving tyrosine *vs *phenylalanine (Tyr→Phe). These amino acid substitutions, however, may not directly affect the potential peptide binding affinity to the HLA-A*0201 molecule (Table 4).

**Table 4:** Blast alignment of LTag 579 in polyomavirus strains

**Polyomavirus strains**	**LTag 579–587**	**Aminoacid substitutions**
BKV (14 strains)	LLLIWFRPV	none
BKV (1 strain)	LLLIW(S)RPV	Ser → Phe; position 6
JCV (155 strains)	LLLIWFRPV	none
JCV (1 strain)	LLLIWF(K)PV	Lys → Arg; position 7
SV40 (13 strains)	L(M)LIW(Y)RPV	Met → Leu and Tyr → Phe; positions 2,6

Most importantly, LTag 579 peptide showed no homology beyond LTag of polyomaviruses, thus indicating that the strong immune responsiveness detected cannot be attributed to cross-reactivities against unrelated highly immunogenic determinants (data not shown)[37].

## Discussion

Polyomavirus large tumor-antigen (LTag) interacts with a number of host molecules involved with cell cycle, including the tumor-suppressor gene product p53 and has been identified as an important target of cancer immunity in murine models[38]. Early studies in SV40 infected mice have reported on conserved CD8+ T cells immune responses against SV40 LTag[39]. In humans, however, despite continuous efforts aimed at identifying potential HLA class I restricted LTag epitopes[40,41], an epitope-specific cytotoxic immune response against this antigens or its portions is still poorly characterized [42,43].

Upon viral entry in non-permissive cells, in the context of an abortive infection, viral DNA is fully integrated in the host genome or resides in host cells as plasmid, thereby favouring the persistence of the virus without production of progeny virions[8]. Within these modalities of virus-cell interaction, LTag is the most highly expressed BKV antigen in non permissive infected cells and might encompass viral epitopes generated by the endogenous class I restricted antigen processing pathway and, possibly, targeted by specific CTL responses. Thus, the identification of LTag derived antigenic peptides might provide decisive advances for the analysis of BKV specific immune responses against infected non permissive cells.

Importantly, LTag regions required for viral transformation, including pRb domains aa101–118; p53 domains aa351–450 and aa533–626, accounting for about 80% of the entire sequence, are less likely to be mutated or lost, and are thus highly genetically conserved[44].

HLA-class I restricted immune responses against defined viral antigens can be frequently induced by ex vivo stimulation of peptide-specific T cells from seropositive subjects[45]. Taking advantage of well studied algorithms, in this study we comprehensively analysed CTL responses against BKV LTag-related epitopes in HLA-A*0201+ BKV LTag experienced individuals. In particular, we focused on antigen triggered cytokine gene expression and lytic capacity in BKV specific CTL.

A first important result of our study is represented by the validation of a rapid, previously characterized[31], screening technology. Indeed, we found a highly significant correlation between IFN-γ gene expression induced by a 3-hour stimulation of CD8+ T cells and the corresponding epitope specific cytotoxic activity detectable following 2–3 week cultures in the presence of peptides and IL-2.

Most interestingly, since antigen triggered cytokine gene expression is detectable after a 3-hour culture only, it is unlikely to be related to primary "in vitro" sensitization, but rather to the stimulation of antigen experienced specific CD8+ T cells, present in the peripheral blood samples under investigation[46]. Moreover, this pattern of epitope immunorecognition "ex vivo" and following "in vitro" culture was consistently reproducible in serial tests performed on cells from the same donors for over one year, thus suggesting that these sustained responses are not associated to specific phases of the host-virus interaction.

LTag 406, 410 and 579 emerged as the most immunogenic peptides among those studied. The latter appeared to be naturally processed in non professional HLA-A*0201 positive antigen presenting cells expressing LTag. The relatively weak response detected following co-cultures of peptide specific expanded T cells with LTag transfected targets (only 3 of 5 cultures produced detectable levels of IFN-γ following stimulation) could be ascribed either to: i) low expression of LTag protein (in spite the relevant production of LTag mRNA following transient transfection) and subsequent impaired peptide-processing; or ii) T cell anergy due to the lack of professional antigen presenting cells (i.e. mDC).

Four out of five HLA-A*0201+ BKV LTag seropositive donors responded to LTag 579 and LDA data indicate that the frequency of CTLp specific for this peptide may be comparable to that of CTLp specific for the highly immunogenic HLA-A*0201-restricted influenza matrix 58–66 epitope (Michel Adamina, manuscript in preparation), as detectable in seropositive individuals.

CTL specific for antigens from viruses responsible for persistent infections are frequently characterized by specific phenotypic profiles. For instance, EBV or CMV specific CTL responses are elicited by distinct subsets of memory T lymphocytes reported as early effectors for EBV (CD45RA-(+)/CCR7+) or late effectors for CMV (CD45RA+/CCR7-)[47]. Our studies indicate that BKV LTag 579 specific CTL responders exquisitely belong to a CD45RA+/CCR7+(-) CD8+ T cell population. Indeed "ex vivo" studies clearly demonstrate CD8+ T cells characterized by a relatively dim positivity for LTag 579/HLA-A*0201 pentamers and displaying this phenotypic profile in the peripheral blood of BKV seropositive donors. In addition 2–3 week cultures of separated cell populations indicate that specific CTL can only be generated from CD45RA+ cells. Importantly, even after these culture times, LTag 579 specific CD8+ T cells retain a CD45RA+/CCR7- phenotype, a relatively infrequent event for CTL recognizing BKV unrelated peptides.

Our study demonstrates for the first time that virtually all HLA-A*0201+ BKV seropositive donors mount a powerful CTL response towards epitopes encompassed by a highly phylogenetically conserved region of the LTag implicated in the viral replicative activity and in the p53 mediated control of the cell cycle of host cells.

These results set the stage for further research aiming at identifying appropriate formulations of LTag immunogenic peptides that might be used to stimulate BKV specific CTL responses in patients at risk of infection reactivation and at clarifying whether this specific immunosurveillance fails in patients bearing tumors potentially associated to BKV mediated oncogenic transformation.

Interestingly, these results may possibly also be extended to JCV-seropositive individuals, as already seen for VP1[48], due to the high homology that the two LTag-p53 binding domains share among the human polyomavirus BK and JC strains. Clearly, in this context, our data may suggest combined use of epitopes from capsid proteins and LTag leading to innovative approaches in the treatment and prevention of human polyomavirus related diseases.

## Abbreviations

BKV, Polyomavirus BK; JCV, Polyomavirus JC; SV40, Simian virus 40; LTag, Large Tumor antigen; VP1, Viral Capsid protein 1; EGFP, Enhanced Green Fluorescent Protein; CTLp, Cytotoxic T Lymphocyte precursor; FI, Fluorescence Index

## Competing interests

The author(s) have no financial conflict of interest.
